# COMP report: CPQR technical quality control guideline for medical linear accelerators and multileaf collimators

**DOI:** 10.1002/acm2.12236

**Published:** 2017-12-04

**Authors:** Charles Kirkby, Esmaeel Ghasroddashti, Crystal Plume Angers, Grace Zeng, Erin Barnett

**Affiliations:** ^1^ Department of Medical Physics Jack Ady Cancer Centre Lethbridge Alberta Canada; ^2^ Department of Oncology University of Calgary Calgary Alberta Canada; ^3^ Department of Physics and Astronomy University of Calgary Calgary Alberta Canada; ^4^ Department of Medical Physics Ottawa Hospital Cancer Centre Ottawa Ontario Canada; ^5^ Department of Medical Physics Mississauga Halton/Central West Regional Cancer Program Trillium Health Partners Mississauga Ontario Canada; ^6^ Department of Medical Physics Stronach Regional Cancer Centre Southlake Regional Health Centre Newmarket Ontario Canada

**Keywords:** linear accelerator, multileaf collimator, radiotherapy quality assurance

## Abstract

The Canadian Organization of Medical Physicists (COMP), in close partnership with the Canadian Partnership for Quality Radiotherapy (CPQR) has developed a series of Technical Quality Control (TQC) guidelines for radiation treatment equipment. These guidelines outline the performance objectives that equipment should meet in order to ensure an acceptable level of radiation treatment quality. The TQC guidelines have been rigorously reviewed and field tested in a variety of Canadian radiation treatment facilities. The development process enables rapid review and update to keep the guidelines current with changes in technology (the most updated version of this guideline can be found on the CPQR website). This particular TQC details recommended quality control testing for medical linear accelerators and multileaf collimators.

## 1 INTRODUCTION

The Canadian Partnership for Quality Radiotherapy (CPQR) is an alliance amongst the three key national professional organizations involved in the delivery of radiation treatment in Canada: the Canadian Association of Radiation Oncology (CARO), the Canadian Organization of Medical Physicists (COMP), and the Canadian Association of Medical Radiation Technologists (CAMRT). Financial and strategic backing is provided by the federal government through the Canadian Partnership Against Cancer (CPAC), a national resource for advancing cancer prevention and treatment. The mandate of the CPQR is to support the universal availability of high quality and safe radiotherapy for all Canadians through system performance improvement and the development of consensus‐based guidelines and indicators to aid in radiation treatment program development and evaluation.

This document contains detailed performance objectives and safety criteria for *Medical Linear Accelerators and Multileaf Collimators*. The development of the individual TQC guidelines is spearheaded by expert reviewers and involves broad stakeholder input from the medical physics and radiation oncology community.^1^ Refer to the overarching document *Technical Quality Control Guidelines for Canadian Radiation Treatment Centres* 2 for a programmatic overview of technical quality control, and a description of how the performance objectives and criteria listed in this document should be interpreted.

All information contained in this document is intended to be used at the discretion of each individual center to help guide quality and safety program improvement. There are no legal standards supporting this document; specific federal or provincial regulations and license conditions take precedence over the content of this document.

## 2 SYSTEM DESCRIPTION

Medical linear accelerators (linacs) are cyclic accelerators which accelerate electrons to kinetic energies from 4 MeV to 25 MeV, using nonconservative microwave radio frequency (RF) fields in the frequency range from 10^3^ MHz (L band) to ~10^4^ MHz (X band), with the vast majority running at 2856 MHz (S band).^3^, ^4^, ^5^, ^6^ In a linear accelerator the electrons are accelerated following straight trajectories in special evacuated structures called accelerating waveguides. Electrons follow a linear path through the same, relatively low potential difference several times; hence, linacs also fall into the class of cyclic accelerators just like the other cyclic machines that provide curved paths for the accelerated particles (e.g., betatrons). The high power RF fields used for electron acceleration in the accelerating waveguides are produced through the process of decelerating electrons in retarding potentials in special evacuated devices called magnetrons or klystrons.

Various types of linacs are available for clinical use. Some provide X‐rays only in the low megavoltage range (4 MV or 6 MV) while others provide both X‐rays and electrons at various megavoltage energies. A typical modern high‐energy linac will provide two or three photon energies (usually a combination of a low [4 to 10 MV] and a high [12 to 25 MV] photon beam) and several electron energies (ranging from 4 to 22 MeV).

Included in the scope of this document are multileaf collimators (MLCs); computer‐controlled devices capable of providing photon beam shielding for linear accelerators using high density leaves (typically tungsten alloy) which are projected into the radiation field.^7^, ^8^, ^9^ In addition to static beam shaping, beam intensity modulation can also be achieved by adjusting the position of the MLC in the radiation field between treatment fields (step and shoot, or static intensity‐modulated radiation therapy [IMRT]), by moving the leaves across the field with varying velocities during the beam‐on time (dynamic IMRT), or by varying the dose rate, gantry speed, and MLC leaf positions during arc delivery (volumetric modulated arc therapy [VMAT]). By doing this, a desired fluence pattern can be approximated within certain physical limits.

Current MLC systems vary with respect to design, location, and use. They may be installed as a tertiary device below the secondary collimators, or they may comprise a total or partial replacement of the secondary collimators. The leaves must provide an acceptable degree of beam attenuation, provide a large enough field coverage, and must be well integrated with the rest of the collimator shaping system. In order to minimize penumbra, various design considerations have been devised by manufacturers to provide focused field shaping.

Computer control is a key component of the MLC, particularly during the delivery of dynamic treatments. There must be feedback on the leaf position and beam interlock capabilities when leaf misplacement is detected. In addition, there must be interlock capabilities to detect leaf carriage positions that could lead to unintentional irradiation outside the shielded area. Other safety interlocks must recognize the unintentional use of the MLC in electron mode and incorporate the use of the MLC in port‐film mode (Tables 1, 2, 31–3).

**Table 1:** Daily quality control tests

Designator	Test	Performance	
		Tolerance	Action
Daily			
DL1	Motion interlock	Functional	
DL2	Couch brakes	Functional	
DL3	Beam interrupt/counters	Functional	
DL4	Lasers/crosshairs	1 mm	2 mm
DL5	Optical distance indicator	1 mm	2 mm
DL6	Optical back pointer	2 mm	3 mm
DL7	Field definition: Jaws/MLC leaves	1 mm	2 mm
DL8	Output constancy – photons	2%	3%
DL9	Output constancy – electrons	2%	3%
DL10	Dynamic (Varian), Virtual (Siemens) or Universal (Elekta) wedge factors	2%	3%

**Table 2:** Monthly quality control tests

Designator	Test	Performance	
		Tolerance	Action
Monthly			
ML1	Wedge, tray, cone, interlocks	Functional	
ML2	Accessories integrity and centering	Functional	
ML3	Gantry angle readouts	0.5°	1.0°
ML4	Collimator angle readouts	0.5°	1.0°
ML5	Crosshairs centering/collimator rotation isocenter (mechanical)	1 mm	2 mm
ML6	Couch position readouts	1 mm	2 mm
ML7	Couch rotation isocenter (mechanical)	1 mm	2 mm
ML8	Couch isocentric angle	0.5°	1.0°
ML9	Optical distance indicator	1 mm	2 mm
ML10	Relative dosimetry	2%	3%
ML11	Central axis depth dose reproducibility	1%/2 mm	2%/3 mm
ML12	Beam profile constancy	2%	3%
ML13	Light/radiation coincidence	1 mm	2 mm
ML14	Jaw position accuracy	1 mm	2 mm
ML15	Backup jaw position accuracy (Elekta)	1 mm	2 mm
ML16	MLC leaf position accuracy	1 mm	2 mm
ML17	Dynamic leaf position accuracy (picket fence)	0.5 mm	1 mm
ML18	Dynamic MLC fluence delivery	95% ≤ 3%/3 mm	95% ≤ 5%/3 mm
ML19	Variation in dose rate, gantry speed, MLC leaf speed and position during arc delivery	See note: ML19	See note: ML19
ML20	Records	Complete	

**Table 3:** Annual quality control tests

Designator	Test	Performance	
		Tolerance	Action
Annual			
AL1	Profile reproducibility	2%	3%
AL2	Depth dose reproducibility	1%	2%
AL3	Reference dosimetry	1%	2%
AL4	Relative output factor reproducibility	1%	2%
AL5	Wedge transmission factor reproducibility	1%	2%
AL6	Accessory transmission factor reproducibility	1%	2%
AL7	Wedge profile reproducibility	1%	2%
AL8	Profile and output reproducibility versus gantry angle	1%	2%
AL9	Monitor chamber linearity	1%/1 MU	2%/2 MU
AL10	End monitor effect	0.5 MU	1 MU
AL11	Collimator rotation isocenter (radiation)	1 mm	2 mm
AL12	Gantry rotation isocenter (mechanical and radiation)	1 mm	2 mm
AL13	Couch rotation isocenter (radiation)	1 mm	2 mm
AL14	Coincidence of radiation and mechanical isocenters	1 mm	2 mm
AL15	Coincidence of axes of rotation	1 mm	2 mm
AL16	Couch deflection	3 mm	5 mm
AL17	Leaf transmission (all energies)	1%	2%
AL18	Leakage between leaves (all energies)	2%	3%
AL19	Transmission through abutting leaves	2%	3%
A20	MLC leaf alignment with jaws	0.5°	1°
A21	Dosimetric leaf gap	0.2 mm	0.3 mm
AL22	Independent quality control review	Complete	

## 3 RELATED TECHNICAL QUALITY CONTROL GUIDELINES

In order to comprehensively assess medical linear accelerator performance, additional guideline tests, as outlined in related CPQR Technical Quality Control (TQC) guidelines must also be completed and documented, as applicable. Related TQC guidelines, available from: http://www.cpqr.ca/programs/technical-quality-control/, include:

- Safety Systems^10^
- Major Dosimetry Equipment^11^
- Accelerator‐integrated Cone‐beam Systems for Verification Imaging^12^
- Patient‐specific Dosimetric Measurements for Modulated Therapies^13^

## 4 TEST TABLES

To ensure a safe and acceptable level of radiation treatment quality the performance of medical linear accelerators and their associated multileaf collimators must be assessed and monitored as a part of a comprehensive quality control program. Tables one through three, below, along with their associated notes, summarize the tests, frequencies, tolerances and action levels recommended for this equipment within such a program.

### Notes on daily tests

**Table:** 

DL1	This test establishes that motion‐enabling features on the linac (e.g., those that allow the gantry to rotate only under desired conditions) are operational. These include functionality tests of couch and hand‐pendant controls and the proper engagement of collision interlocks when touch guards are engaged
DL2	A functional test is performed to establish that brakes on the treatment couch engage when desired and prevent the couch from floating freely or moving when a small force is applied
DL3	This test demonstrates (when applicable): the key interlock prevents the linac from irradiating; the nonemergency beam interruption system stops the beam; upon beam interruption the monitor unitis recorded are in agreement with the backup monitor unit counter and/or timer if applicable
DL4	This test establishes the alignment of crosshairs with appropriate lasers are within the specified limits
DL5	At gantry angle 0°, the test demonstrates that the optical distance indicator identifies the isocenter plane within the specified limits
DL6	This test verifies the performance accuracy of the optical back pointer for applicable units
DL7	Gantry angle 0°, 100 cm source‐axis distance (SAD). This test demonstrates the field edges are accurately defined by jaws and/or MLC leaves. It is sufficient to confirm a predefined field shape using the projected light field at isocenter. Tolerance and action levels apply to each edge of a rectangular field at isocenter as defined by the jaws/MLC leaves. Note that systems with a tertiary collimation MLC system will require both jaw and MLC leaf positions to be verified
DL8	Output constancy must be verified for all photon energies in use on the particular treatment day. Measurement is to be conducted using standard local geometry using a dosimetry system calibrated against the local secondary standard system
DL9	Output constancy must be verified for all electron energies in use on the particular treatment day. Measurement is to be conducted using standard local geometry using a dosimetry system calibrated against the local secondary standard system
DL10	Wedge factors for a representative set of dynamic or virtual soft wedges in use on a particular treatment day must be verified. Machine design characteristics must be considered when determining the representative set. Alternatively, a test cycle designed to test the full range of wedges over multiple days may be considered. Daily wedge factors for universal wedges are required to ensure functionality and position reproducibility

### Notes on monthly tests

**Table:** 

ML1	Verify the functionality of latching interlocks (includes verification that electron beams cannot be turned on unless the MLC leaves are retracted)
ML2	Verify the physical integrity and centering of accessories, including wedges, trays, and cones, as appropriate
ML3	The accuracy of the digital and mechanical (if used clinically) gantry angle readouts must be verified for at least 0°, 90°, 180°, and 270°. The coordinate system convention should also be verified
ML4	The accuracy of the digital and mechanical (if used clinically) collimator angle readouts must be verified for at least 0°, 90°, 180°, and 270°. The coordinate system convention should also be verified
ML5	This test establishes the correct centering of the crosshairs as well as the mechanical axis of rotation of the collimator. Tolerance and action levels refer to the maximum diameter of the mechanical isocenter and the maximum displacement of the crosshairs projection from the center of the mechanical isocenter circle
ML6	Mechanical and digital couch position readouts must be verified over an appropriate clinical range in the directions of the three cardinal axes. Also verify coordinate system convention
ML7	Isocentric rotation of the couch about the collimator rotation axis must be verified. Similar to ML5, the tolerance and action levels refer to the maximum displacement of crosshairs projection from the initial position in the isocenter plane
ML8	Mechanical and digital couch isocentric rotation angle readouts must be verified over the applicable clinical range. Also verify coordinate system convention
ML9	A mechanical device, calibrated against the true radiation isocenter, is used to provide the base reading for the check of the optical distance indicator. The standards stated in the Table apply at the isocenter. The optical distance indicator should be checked over a clinically relevant range of source‐to‐skin distances (SSDs) and gantry angles. The tolerance and action levels may be twice as large (i.e., 2 mm and 4 mm) at the clinical limits of the optical distance indicator's range
ML10	Using a dosimetry system calibrated against the local secondary standard, the output of all clinical beams is checked against yearly reference dosimetry
ML11	Measurements are made to confirm that the depth dose has not changed since commissioning the unit. Tolerance and action levels are specified in percentages for photon beams and in millimetres for electron beams. A single ratio of doses taken at clinically relevant depths is sufficient for these measurements. Alternatively, a tissue‐phantom ratio (TPR) measurement or a check of profile constancy at a shallow depth could be used, and the tolerance and action levels adjusted appropriately
ML12	This test replaces testing of flatness and symmetry and is intended to be consistent with the testing suggested in American Association of Physicists in Medicine (AAPM) protocol TG‐142.^14^ The goal is to ensure that profiles are delivered in a manner consistent with that modeled in the associated treatment planning system. Tolerance and action levels refer to differences from commissioning (or baseline) profiles as defined in the AAPM protocol TG‐142.^14^ Separate tests are required for all clinically applicable beams
ML13	Geometric alignment of the radiation and optical field edges must be established over a range of field sizes. Tolerance and action levels apply to each edge of a rectangular field
ML14	Accuracy of the radiation field edge of the jaw must be established over a range of jaw positions. The number of positions tested shall be determined from the jaw calibration method. In conjunction with this test it is important to establish acceptable dose profiles for abutting fields at the 0 position. Here, the 2 mm action level for each jaw is generally not sufficient since in principle, abutting fields could have a difference of up to 4 mm between field edges, which can lead to unacceptable peaks or valleys in dose distributions. A tolerance of 5% and an action level of 10% in dose profile deviations for abutting fields are suggested
ML15	Accuracy of the radiation field edge of the backup jaw must be established over a range of positions, if applicable. The number of positions tested shall be determined from the jaw calibration method
ML16	Accuracy of the radiation field edge of the MLC leaf edges must be established over a range of MLC positions. The number of MLC positions tested shall be determined from the MLC calibration method. For some MLC designs this test may be accomplished by evaluating the radiation position of each leaf relative to a reference leaf
ML17	For dynamic MLC IMRT, leaf gap accuracy for all leaf pairs is verified via inspection of a two‐dimensional dose map of a picket fence pattern delivered at gantry angle of 0°
ML18	Specific to IMRT, this test demonstrates that the interplay of leaf velocity, gap width, gap position, and beam holds combine to deliver a planar dose map consistent with the prediction of the treatment planning system. A test plan should consider extreme conditions (e.g., the highest levels of modulation used clinically for each leaf pair). An acceptable alternative to this test is the regular (more than once per month) measurement of patient‐specific, dynamic MLC IMRT fields. Tolerance and action levels are defined via the gamma metric comparing dose map differences (plan versus measurement). Dose maps are defined with region of interest threshold of 10% of the maximum dose. Dose differences are global (i.e., with respect to maximum dose).^14^ Detector resolution must be sufficient to identify performance of individual leaves. As with all tests, tolerance and action levels may be tightened at the user's discretion
ML19	The synchronicity of all dynamic parameters during arc delivery is verified. Parameters may be evaluated independently, using a subset of the tests described by Ling et al.^15^ or Bedford and Warrington,^16^ or by the repeat delivery of a standard VMAT plan of suitable complexity, similar to test ML18. Tolerance and action levels are in reference to the consistency of dose delivered at different dose rate, gantry or MLC speeds. Tolerance levels should be based on the performance of the linear accelerator, whereas action levels should be set to achieve an overall precision consistent with other monthly tests (approximately 3%/2 mm from baseline)
ML20	Documentation relating to the daily quality control checks, preventive maintenance, service calls, and subsequent checks must be complete, legible, and the operator identified

### Notes on annual tests

**Table:** 

AL1	This test establishes that an appropriate subset of the cross‐plane and in‐plane profiles at gantry angle 0° are consistent with water‐tank measurements made at the time of commissioning. Tolerance and action levels refer to differences from commissioning or baseline. Measurements should be made for all clinically operable beams.
AL2	Depth dose scans necessary for calibration protocols (alternatively TPR measurements) are also made and used to verify consistency with commissioning/baseline water‐tank measurements. Tolerance and action levels refer to differences from commissioning or baseline. Measurements should be made for all clinically operable beams
AL3	A full absolute dosimetry output calibration based on an internationally accepted protocol (e.g., AAPM TG‐51)^17^ must be performed annually on each energy used clinically for both photons and electrons. Independence of output with respect to dose rate (pulse repetition frequency) must also be established across clinically applicable dose rates
AL4	An appropriate subset of relative output factors are confirmed to be consistent with commissioning measurements
AL5	The wedge transmission factors (if applicable) are confirmed to be consistent with commissioning measurements
AL6	Transmission factors are confirmed to be consistent with commissioning measurements. Discretion may be used. Devices where the physical composition/dimension can be confirmed not to have changed since a previous measurement need not be measured again
AL7	This test applies to moving jaw (dynamic and virtual) and universal (Elekta) wedges. This test confirms that wedged fields produce profiles that are consistent with baseline data through the central 80% of the field for all clinically used wedge angles
AL8	This test establishes the independence of output with gantry angle. It requires that output be measured under identical conditions (e.g., dosimeter under the same amount of buildup material in each position) and that the difference from the gantry at 0° position be within the specified limits. In addition to central axis output, beam profiles must be measured at three cardinal gantry angles: 0°, 90°, and 270°. Measurements should be made for all clinically operable beams
AL9, 10	From a series of radiation measurements with different monitor units the linearity and the end monitor effect are determined.^18^ The larger of the percentage or absolute value is taken as what is applicable. Measurements should be made for all clinically operable beams
AL11	Commonly measured using a star shot technique,^19^ this test determines the diameter of the circle that intersects all rays formed by the projection of a narrow field as the collimator is rotated through an appropriate sample of angles within its full range of motion. The diameter must be within specifications
AL12	This test determines the diameter of both the mechanical and the radiation isocenter defined by gantry rotation through its full clinical range of motion. Each diameter must be within specifications
AL13	This test determines the diameter of the radiation isocenter defined by couch rotation through its full clinical range of motion. The diameter must be within specifications
AL14	The coincidence of radiation and mechanical isocenters is established for the collimator, gantry and couch. Coincidence must meet the specified limits
AL15	The three axes of rotation (the collimator/MLC, the couch, and the gantry) must meet within a sphere of the specified diameter.^20^, ^21^, ^22^
AL16	Couch deflection is measured as a difference in surface position (load versus no load) of the couch extended longitudinally at least 30 cm through isocenter. Under “load” is considered as a typical patient mass (approximately 70 kg) distributed over the couch or placed at the center. The difference is the couch deflection. Tolerance and action levels are defined relative to the deflection measured at the time of commissioning
AL17	The average and maximum MLC leaf transmission is verified in this test for all photon energies and compared with the values established at the time of commissioning or the values adopted in the treatment planning system. Tolerance and action levels refer to changes from the commissioning measurements
AL18	The average and maximum leakage between adjacent, closed MLC leaves is verified in this test for all photon energies and compared with the values established at the time of commissioning or the values adopted in the treatment planning system. Tolerance and action levels refer to changes from the commissioning measurements
AL19	The average and maximum leakage between abutting closed MLC leaves is verified in this test for all photon energies and compared with the values established at the time of commissioning or the values adopted in the treatment planning system. Tolerance and action levels refer to changes from the commissioning measurements
AL20	Use a leaf pattern where one leaf from each leaf bank protrudes well into the field. Confirm the leaf edge parallelism with the collimator or solid jaw edge
AL21	A dynamic leaf gap test (sometimes referred to as a dosimetric leaf gap test) is performed to confirm consistency with baseline measurements. The minimum standard is to establish this using a single detector (e.g., an ion chamber) method, although methods that calculate separate factors for each leaf pair may be employed. The value must be consistent within tolerance for all four cardinal gantry angles
AL22	To ensure redundancy and adequate monitoring, a second qualified medical physicist must independently verify the implementation, analysis, and interpretation of the quality control tests at least annually

## CONFLICT OF INTEREST

The authors have no conflicts of interest to declare.
